# An ace-1 gene duplication resorbs the fitness cost associated with resistance in Anopheles gambiae, the main malaria mosquito

**DOI:** 10.1038/srep14529

**Published:** 2015-10-05

**Authors:** Benoît S. Assogba, Luc S. Djogbénou, Pascal Milesi, Arnaud Berthomieu, Julie Perez, Diego Ayala, Fabrice Chandre, Michel Makoutodé, Pierrick Labbé, Mylène Weill

**Affiliations:** 1 CNRS, IRD, ISEM—UMR 5554, Montpellier, France; 2 University of Montpellier, Montpellier, France; 3 IRSP, Ouidah, Benin; 4 University of Abomey-Calavi, Cotonou, Benin; 5 IRD, CNRS, MIVEGEC—UMR 224/5290, Montpellier, France

## Abstract

Widespread resistance to pyrethroids threatens malaria control in Africa. Consequently, several countries switched to carbamates and organophophates insecticides for indoor residual spraying. However, a mutation in the *ace-1* gene conferring resistance to these compounds (*ace-1*^*R*^ allele), is already present. Furthermore, a duplicated allele (*ace-1*^*D*^) recently appeared; characterizing its selective advantage is mandatory to evaluate the threat. Our data revealed that a unique duplication event, pairing a susceptible and a resistant copy of the *ace-1* gene spread through West Africa. Further investigations revealed that, while *ace-1*^*D*^ confers less resistance than *ace-1*^*R*^, the high fitness cost associated with *ace-1*^*R*^ is almost completely suppressed by the duplication for all traits studied. *ace-1* duplication thus represents a permanent heterozygote phenotype, selected, and thus spreading, due to the mosaic nature of mosquito control. It provides malaria mosquito with a new evolutionary path that could hamper resistance management.

Vector-borne diseases, among which malaria is preeminent, cause a considerable burden on human populations^1^. In sub-Saharan Africa, *An. gambiae* is the major malaria vector. Malaria vaccine is still under experimentation and access to anti-malaria drugs remains difficult and expensive, thus mosquito vectors control is the only affordable measure to fight malaria^2^,^3^. Mosquito control worldwide relies essentially on the use of chemical synthetic insecticides that target an insects’ vital function^4^. Only four classes of conventional insecticides are licensed by the World Health Organization (WHO): Organochlorines (OCs), Pyrethroids (PYRs), Carbamates (CXs) and Organophosphates (OPs)^5^. Direct control of *Anopheles* breeding sites is usually not possible, and the main option to block or reduce malaria transmission is to prevent the vector-host contact using insecticide-treated bed-nets (ITNs) and indoor residual house spraying (IRS).

Until recently, PYRs were the only insecticides authorized for ITNs and the most used for IRS^4^,^6^,^7^,^8^,^9^,^10^,^11^,^12^. These tools have been shown to efficiently protect vulnerable populations from endemic countries^1^,^13^. Unfortunately, due to large-scale and prolonged treatments, as well as mosquito populations large effective size and their short life span per generation, resistance to PYRs was rapidly selected, and is now widespread in most malaria vectors from sub-Saharan Africa^14^,^15^. Several alarming studies predicted that PYR resistance may contribute to malaria vector control failure^15^,^16^,^17^,^18^.

In order to preserve vector control effectiveness, alternative solutions to PYRs are urgently needed. However, with the limited number of insecticides and none expected in the near future^14^, OPs and CXs were suggested as potential alternative compounds to control PYR-resistant populations, either alone or in combination with PYRs^17^,^19^,^20^,^21^. They have indeed shown a good efficacy in ITNs and IRS, with high mortality of PYR- resistant (*kdr*^*R*^) *An. gambiae* in Ivory Coast and Benin^22^,^23^,^24^. Thus, following the American President’s Malaria Initiative in collaboration with the National Malaria Control Program, several African countries recently switched partly or entirely from PYRs to CXs (i.e. bendiocarb) or OPs (i.e. chlorpyrifos or pirimiphos methyl) for IRS^18^,^22^,^25^,^26^.

However, a particular concern for the use of OPs and CXs is that resistance to these insecticides is already present in some *An. gambiae* populations from West Africa^27^,^28^,^29^,^30^. Although resistance has also been shown to result from overexpression of detoxification enzymes^31^,^32^, highest resistance levels are due to mutation in the target of OPs and CXs, the acetylcholinesterase (AChE1) encoded by the *ace-1* gene: a single amino acid substitution of glycine by serine at the position 119 (G119S) resulting in a major conformational change^33^. This *ace-1*^*R*^ resistant allele arose independently several times in distinct mosquito species^34^,^35^. In *Culex pipiens* mosquitoes, it entails a large fitness cost for several life history traits^36^,^37^,^38^. A similar fitness cost appears to exist for *An. gambiae* (a single study showed that pupae carrying *ace-1*^*R*^ endure higher mortality rate^39^). Thus, while resistant mosquitoes survive in the presence of insecticide, they are outcompeted by susceptible in absence of insecticide, due to their lower fitness. This fitness cost is crucial for resistance management: in absence of OPs and CXs selective pressures, *ace-1*^*R*^ frequency should indeed decrease (the costlier the faster^40^), allowing insecticide rotation or mosaic strategies to maintain low levels of insecticide resistance.

Worryingly, a new *ace-1* allele has been found in *An. gambiae* and *An. coluzzii* in several West African countries (e.g. Ivory Coast and Burkina Faso)^41^,^42^. This allele, named *ace-1*^*D*^, consists in a duplication of the *ace-1* gene, associating a susceptible and a resistant copy probably on the same chromosome. Several similar duplicated alleles have been observed in *Cx. pipiens*^43^,^44^, where they have been shown to be selected because they reduce the fitness cost associated with the G119S mutation^45^,^46^,^47^. A selective advantage was also recently described in *Drosophila melanogaster* with the duplication of the resistance target gene *Rdl*^*R*^^48^. A similar selective advantage of *An. gambiae ace-1*^*D*^ allele would facilitate its diffusion in natural populations, thereby spreading OPs and CXs low-cost resistance and endangering malaria vector control strategies. It is thus crucial to evaluate the threat of this *ace-1* duplication by investigating its impacts on the fitness of *An. gambiae*, both in presence and absence of insecticide.

To do so, we constructed a laboratory strain homozygous for the *ace-1*^*D*^ allele and sharing a genetic background similar to the reference strains KisumuP and Acerkis, respectively homozygous for the single-copy susceptible *ace-1*^*S*^ and resistant *ace-1*^*R*^ alleles, a mandatory step to avoid any confounding effect due to other resistance mechanisms or any other mutations. We analyzed the organization of the duplicated *ace-1* gene by a cytogenetic approach, and compared the three strains performances for OPs and CXs resistance levels, as well as several life history traits. This study revealed that *ace-1*^*D*^ is indeed expected to spread, threatening the switch to OPs or CXs for malaria mosquito control in countries with PYR-resistant populations.

## Results

### Characterization of the Acerduplikis strain carrying *ace-1* gene duplication

We collected larvae from a wild population of *An. gambiae* in Baguida^49^, a region suspected to contain *ace-1*^*D*^ alleles, because of a large apparent excess of heterozygous [RS] phenotypes at the *ace-1* locus^42^. As no enzymatic or molecular test is currently available for detecting *ace-1* duplication, we used the genetic protocol developed by Labbé *et al.*^44^ for *Cx. pipiens* mosquitoes, to identify females harboring *ace-1*^*D*^ alleles (Supp. Fig. 1). The *ace-1*^*D*^ allele is composed of susceptible, D(S), and resistant, D(R), copies (see nomenclature in Labbé *et al.*^43^). They were sequenced in 16 females (2241 bp PCR fragment from exon 2 to exon 7), and all D(S) copies were found identical, as were all D(R) copies. The D(R) copy is strictly identical to the known *ace-1*^*R*^ allele^32^ and differs from the D(S) copy by 24 mutations (Supp. Fig. 2). Both D(S) and D(R) copies were found identical to the sequence of the *ace-1*^*D*^ allele previously detected in *An. gambiae* species^41^. Progenies of the 16 founding females were mixed to construct the Acerduplikis strain.

The number of *ace-1* gene copies was estimated for 20 Acerduplikis and KisumuP mosquitoes with Real-time quantitative PCR. Differences in copy number among D/D and S/S genotypes was tested by computing the following linear model Cn = Geno + ε, with Cn the copy number, Geno the genotype (S/S or D/D) of each individual and ε the error parameter (Gaussian distribution). It confirmed that Acerduplikis significantly displays twice as much *ace-1* copies (2.13 ± 0.27) as KisumuP (1.00 ± 0.05; LRT, *F* = 329.9, *Δdf* = 1, *p* < 0.001, Supp. Fig. 3).

**Figure 1:**
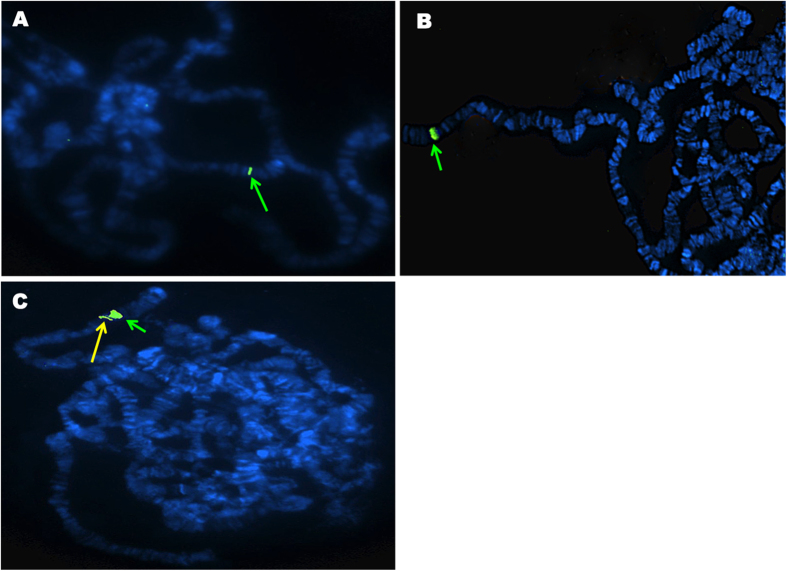
*In situ* hybridization with Cy3 fluorescently labelled DNA probes performed on polytene chromosomes of *An. gambiae* strains. Green and yellow arrows indicate *ace-1* and AGAP001373 probes respectively. (**A**) *ace-1* probe hybridized on KisumuP strain; (**B**) *ace-1* probe hybridized on Acerduplikis strain; (**C**) *ace-1* and AGAP001373 probes co-hybridized on Acerduplikis strain.

A fluorescence *in situ* hybridization (FISH) approach was used to localize the positions of the two *ace-1* D(S) and D(R) copies of Acerduplikis *ace-1*^*D*^ allele on the chromosomes. Two fluorescent probes were used, *ace-1* and AGAP001373 (probe 2), which are separated by about 500 kb on the 2R chromosome arm of *An. gambiae.* Our results showed a single signal with the *ace-1* probe, at the same location for both KisumuP and Acerduplikis strains (Fig. 1A,B). However, the signal was broader for Acerduplikis. When the two probes were co-hybridized on Acerduplikis polytene chromosomes, we observed two different signals, the broadest corresponding to the *ace-1* probe and the thinner to probe 2 (Fig. 1C). This result evidenced that the two copies of the *ace-1* duplicated allele are in tandem and separated by a distance lower than 500 kb.

Finally, since we aimed at determining the impact of *ace-1*^*D*^ allele on mosquito fitness, we performed eight successive backcrosses to introgress this allele into the susceptible KisumuP reference strain genetic background. Polymorphic sequence markers that differentiate KisumuP and Baguida (a mix of 10 individuals from the Baguida field population used to establish Acerduplikis) were developed on each chromosome (Supplementary Table 1). The Acerduplikis fixed strain shared all the KisumuP markers, showing that the Acerduplikis genomic background was largely similar to that of KisumuP. Although recombination around the *ace-1* gene is not total, most of the background effects were eliminated, allowing a pertinent assessment of the duplication effects on fitness.

### *ace-1* ^*D*^ provides less resistance to carbamates and organophosphates insecticides than *ace-1* ^*R*^

Bioassays were carried out on larvae from the three strains KisumuP (S/S), Acerkis (R/R) and Acerduplikis (D/D) strains and from their F1 offspring (R/S, D/S and D/R genotypes). One CX (bendiocarb), three OPs (chlorpyrifos methyl, fenitrothion and dichlorvos) and one PYR (permethrin) were tested. For all larval bioassays, mortality in control tests never exceeded 5%. Statistical analyses (chi-square test between observed and expected dead numbers) indicated good fits for the log-dose-mortality regressions (all *p-value* > 0.05, Table 1, Supp. Fig. 4). Moreover, the same susceptibility to permethrin was recorded for KisumuP, Acerkis (RR_50_ = 1, *p* > 0.05) and Acerduplikis (RR_50_ = 1, *p* > 0.05) showing the absence of pyrethroid resistance mechanism (Table 1). This last result confirmed that only *ace-1* contributed to OPs and CXs resistance in the tested strains.

The Acerduplikis strain (D/D) displayed a significantly lower resistance level to CX (bendiocarb, RR_50_ = 3.14 *vs* 229.3, *p* < 0.001) and OPs (chlorpyriphos-methyl, RR_50_ = 1.91 *vs* 9.03, *p* < 0.001; fenitrothion, RR_50_ = 6.56 *versus* 23.74, *p* < 0.001; dichlorvos, RR_50_ = 8.78 *vs* 12.61, *p* < 0.001) than Acerkis (R/R; Table 1, Supp. Fig. 4). While D/D individuals displayed a resistance level similar to the R/S heterozygotes for all the tested OPs (all *p-value* > 0.05), and a significantly lower resistance level for the CX bendiocarb (*p* < 0.001). For all the tested insecticides, D/S and D/R heterozygotes displayed, respectively, significantly lower and significantly higher resistance levels (all *p*-values < 0.001) than D/D individuals (Table 1), but D/R individuals displayed significantly lower resistance levels than R/R individuals (all *p*-values < 0.001). From the least to the most resistant, the genotype order is thus: SS <DS <DD ≈RS <DR <RR.

### *ace-1* duplication induces low, if any, fitness cost

To measure the fitness cost associated with the different *ace-1* genotypes, several life history traits were compared in Acerkis (R/R), Acerduplikis (D/D) and KisumuP (S/S).

#### Larval mortality and development time

- *Pre-imaginal mortality* was followed from egg hatching to adult emergence. The number of dead larvae at each developmental stage was recorded, allowing testing for differences between strains in overall mortality as well as in mortality dynamics. A Cox proportional hazards regression model (Cox model) was thus computed as: Surv = Geno + *ε* , with Surv, the proportion of dead larvae at each developmental stage, Geno a three-levels factor corresponding to the different genotypes (S/S, D/D, R/R) and ε the error parameter, following a binomial distribution to take over-dispersion into account, if present. Emerging adults were censored in the analyses.

**Figure 2:**
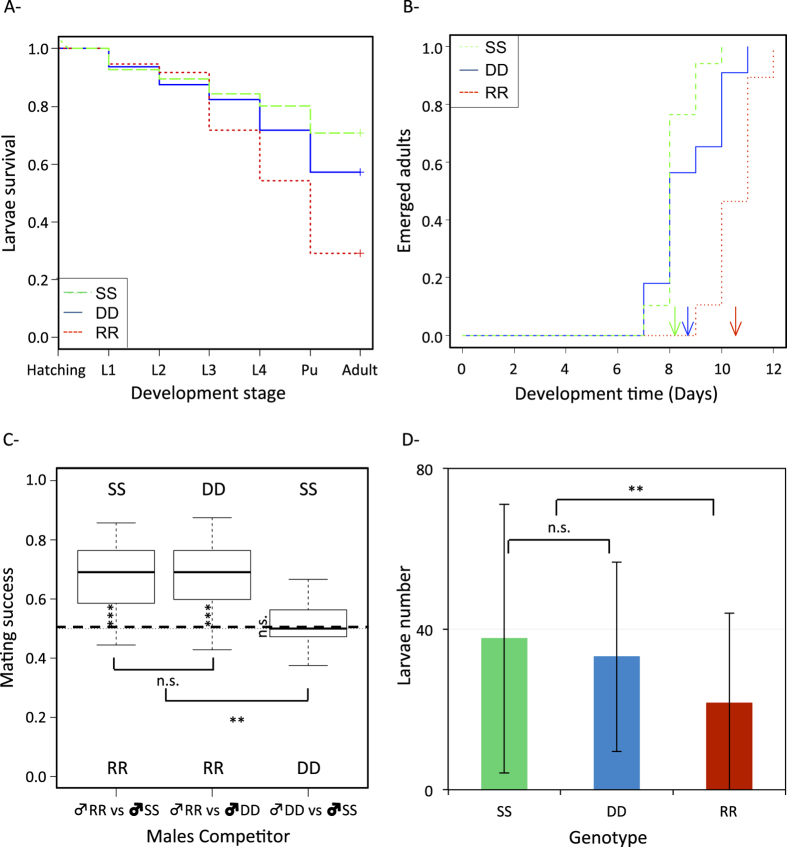
Life history traits of the susceptible KisumuP (SS, green, dash line), resistant Acerkis (RR, red dot line) and resistant duplicated Acerduplikis (DD, blue, solid line) homozygotes. Panel (**A**) *Larval mortality*. The proportion of larvae surviving at each development stage is presented from hatching to emergence (*L*_*i*_ is the larval stage *i* and *Pu* the pupal stage). Crosses represent the proportion of emerged adults. Panel (**B**) *Development time*. The proportion of emerged adults on each day following the experiment beginning is presented for each genotype. Arrows indicate the mean development time of each genotype. Panel (**C**) *Mating competition*. Boxplots present the distribution of paternity success. The horizontal dash line symbolizes an equal paternity success of the two types of males. Significance of the departure from 0.5 is indicated vertically and significance of differences among confrontations is indicated horizontally (n.s., *p* > 0.05; *p < 0.05; **p < 0.01; ***p < 0.001). Panel (**D**) *Female fecundity*. The average larvae numbers by female are presented with their standard deviation. Significance of the differences in fertility is indicated (n.s., *p* > 0.05; *p < 0.05; **p < 0.01; ***p < 0.001).

The duplicated D/D genotype displayed at each larval stage a significantly lower mortality than the R/R genotype (*z* = 3.6, *p* < 0.001). Although it tended to be slightly higher, D/D larval mortality at each stage was not significantly different from the susceptible S/S genotype (*z* = 1.9, *p* = 0.06; Fig. 2A). The overall larval mortalities of each genotype were *m*_*RR*_ = 0.71 [0.60–0.79]; *m*_*DD*_ = 0.43 [0.32–0.52]; *m*_*SS*_ = 0.29 [0.20–0.38] (the 95% confidence intervals, or CI, are given in the brackets).

- *Development time* was recorded as the number of days necessary for a first-instar larva to reach adulthood (i.e. the time until emergence). The sex of each emerging adult was recorded. Differences in development time between genotypes and/or sexes were tested by computing the following Cox model: Dev = Geno + Sex + Geno.Sex + *ε*, with Dev, the number of adults that emerged at a given day, Geno a three-levels factor (S/S, D/D and R/R), Sex a two-levels factor (male or female), Geno.Sex the interaction between the two factors, and ε the error parameter (binomial distribution).

No interaction between sex and genotype was detected (*χ*^*2*^ = 2.22, *Δdf* = 2, *p* = 0.33) allowing studying the impact of each factor independently. As expected in *An. gambiae*^50^, males emerged significantly earlier than females (respectively: 8.5 ± 1.2 and 9.2 ± 1.3 days; *χ*^*2*^ = 8.9, *Δdf* = 1, *p* < 0.01). D/D individuals developed significantly faster than R/R individuals (respectively, 8.7 ± 1.3 and 10.5 ± 0.84; *t* = 7.85, *df* = 76.05, *p* < 0.001). The mean development time was not significantly different between D/D and S/S individuals (respectively, 8.7 ± 1.3 and 8.2 ± 0.7; Student test, *t* = 0.83, *df* = 50.67, *p* = 0.41; Fig. 2B). However, the Cox model showed a larger variance in the development time of D/D than of S/S individuals (i.e. more time between the first and the last adult to emerge; *z* = 3.8, *p* < 0.001; Fig. 2B).

#### Mating competition

Mating competition trials were performed between pairs of males of different genotypes to compare their capacity to inseminate either KisumuP (S/S) or Acerduplikis (D/D) females. A generalized linear model (GLM) was used to compare paternity success among competing male pairs: Pat = Pairs + Fem + Pairs.Fem + ε, with Pat the paternity success (number of egg rafts sired by a given male genotype), Pairs a three-levels factor corresponding to the pairs of male genotypes in the different trials (D/D *vs* S/S, D/D *vs* R/R, and S/S *vs* R/R), Fem a two-levels factor corresponding to the female genotype (S/S or D/D), Pairs.Fem the interaction between these two factors, and ε the error parameter (binomial distribution).

The female genotype did not significantly impact the paternity success, either among the trials (Pairs.Fem: *χ*^*2*^ = 0.07, *Δdf* = 2, *p* = 0.97) or for a given trial (Fem: *χ*^*2*^ = 2.22, *Δdf* = 1, *p* = 0.33). However, the pairs confronted in each trial did not fare similarly (Pairs: *χ*^*2*^ = 11.25, *Δdf* = 2, *p* < 0.01). Both the D/D and the S/S males sired more progenies than R/R males (i.e. paternity success >0.5): in DD *vs* RR trial, D/D paternity success was 0.68 ± 0.11 (>0.5, Binomial test: *p* < 0.001), while in SS *vs* RR trial, S/S paternity success was 0.68 ± 0.12 (>0.5, Binomial test: *p* < 0.001). Paternity successes of the D/D and S/S males were not significantly different, either when confronted to R/R males (*χ*^*2*^ = 0.02, *df* = 1, *p* = 0.89) or to each other: in DD *vs* SS trial, D/D paternity success was 0.48 ± 0.08 (not different from 0.5, Binomial test: *p* = 0.63) (Fig. 2C).

#### Female fecundity and fertility

In order to assess the influence of the duplicated allele on female reproductive success, forty females of each genotype (S/S, D/D and R/R) were allowed to lay eggs. The number of females laying eggs, eggs laid and larvae produced were recorded.

Overall, the reproductive success (Rsuc) of R/R females was significantly lower (on average 21.5 ± 22 larvae per female) than D/D or S/S females (respectively, on average 33.1 ± 24 and 37.6 ± 33 larvae per female; Fig. 2D) (GLM Rsuc = Geno + ε (Gaussian distribution), *F* = 7.04, *Δdf* = 1, *p* < 0.01). Moreover, the difference between D/D and S/S females was not significant (*F* = 0.56, *Δdf* = 1, *p* = 0.46).

The observed differences are due to the fact that R/R females lay fewer eggs than the others; the number of females laying eggs and the hatching rate are not significantly different between the three genotypes. For a detailed analysis see Supplementary materials Supp. Fig. 5.

Overall, the order of the genotypes from the less to the most costly is: SS ≈DD < RR.

## Discussion

As more advisors urge African countries to switch from PYRs to CXs and OPs for malaria vector control, understanding the already-spreading resistance to these insecticides is urgent. The present study contributes by characterizing the impact of the *ace-1* gene duplication on *An. gambiae* fitness. We constructed a laboratory strain homozygous for the *ace-1*^*D*^ allele, sharing a nuclear background similar to the susceptible reference strain, KisumuP, to avoid any confounding effect. We then described this new resistance allele and measured its performances in presence or absence of insecticide compared to single-copy susceptible (*ace-1*^*S*^) and resistant (*ace-1*^*R*^) alleles. Our findings provide clues on how *ace-1*^*D*^ has arisen, but darken the perspectives of using OPs and CXs as alternative to PYRs’ lurking incapacitation due to rising resistance.

### A new resistance allele at the *ace-1* locus

In *Cx. pipiens*, 13 distinct *ace-1* duplicated alleles have been identified so far^44^,^51^,^52^, sometimes with several duplicated alleles in a same population. Although nothing is known about their chromosomal structure, they seem to have arisen from several independent duplication events^43^.

The situation is quite different in *An. gambiae*, as only one *ace-1*^*D*^ allele appears to segregate in West Africa: the unique D(S) and D(R) sequences of the studied allele here and collected in Togo in 2012 are identical to those previously described in Ivory Coast and Burkina Faso in 2006^41^. This strongly suggests that they proceed from the same duplication event. The D(S) and D(R) copies are quite divergent at the nucleotide level, but our study shows that they lay in close tandem on chromosome 2R (Fig. 1). These observations suggest that, among the different scenarios generating duplications^44^, this particular allele probably results from an unequal crossing-over in a standard heterozygote (i.e. R/S).

### *ace-1* duplication sets a new evolutionary path for *Anopheles gambiae* resistance

Current genotyping methods for the *ace-1* locus cannot discriminate *ace-1*^*D*^ carriers from a standard heterozygote. Therefore, *ace-1*^*D*^ frequency in *An. gambiae* natural populations from West Africa has been estimated from the apparent excess of [R/S] phenotypes caused by its presence^42^. Nevertheless, previous estimations suggested that *ace-1*^*D*^ is quite frequent in this region^27^,^42^,^53^. Our study provides the clues to understand the reasons.

Insecticide resistance data showed that the resistance level conferred by the duplicated allele *ace-1*^*D*^ is lower than the one conferred by *ace-1*^*R*^ (Table 1). It also seems correlated to the percentage of R copies carried by mosquitoes: for all tested insecticides, different genotypes resistance order generally as RR >DR >DD ≈RS >DS >SS, similarly to what was previously described in *Cx. pipiens*^46^. A probable explanation could be the competition existing between AChE1S and AChE1R enzymes in the synapse, as increasing the number of S copies will reduce AChE1R enzymes randomly picked from a pool, thereby decreasing the resistance level^45^. A resistance advantage (both in intensity and specificity) of *ace-1*^*D*^ over *ace-1*^*R*^ is thus clearly ruled out.

*An. gambiae* major life history traits analysis (pre-imaginal mortality, larval development time, mating competition, and female fertility) showed that a high fitness cost is associated with the resistant R/R genotype (Fig. 2A–D), confirming the sole previous study available^39^. Moreover, as anticipated^54^, this cost is similar to the one associated with the same G119S mutation in *Cx. pipiens* for several life-history traits in field and laboratory studies^36^,^37^,^45^,^46^: for instance, in this study the pre-imaginal mortality for R/R homozygotes is increased compared to the S/S ~2.57 times in *An. gambiae*, versus ~2.43 times in *Cx pipiens*, (z = 0.97, *p* = 0.33;^46^). The fitness cost associated with *ace-1*^*R*^ was previously attributed to the reduction of insensitive acetylcholinesterase (AChE1R) activity by more than 60% compared to the susceptible one (AChE1S)^55^, a magnitude similar in both mosquito species^54^.

While the existence of such cost for *ace-1*^*R*^ comforts hopes of controlling this resistance allele, our results are grimmer regarding the duplicated allele. The D/D genotype indeed appears less costly than the R/R genotype, to the point that no significant difference was observed between D/D and S/S genotypes on the four life history traits measured (Fig. 2). The D/D genotype performances were nevertheless always slightly lower than S/S ones, which could suggest a low fitness cost. As previously proposed for *Cx. pipiens*^46^, the decreased fitness cost in D/D individuals compared to R/R could result from the reduction of the costly AChE1R relative quantity (as the duplicated allele produces both AChE1R and AChE1S enzymes). To further the comparison between the two mosquito species, the fitness of *An. gambiae ace-1*^*D*^ allele seems to be at least similar to that of the fittest duplicated allele analyzed so far in *Cx. pipiens*, *ace-1*^*D1*^ 45,46. For instance, compared to R/R homozygotes, D_1_/D_1_ pre-imaginal mortality is decreased 1.3 times in *Cx. pipiens*^46^, versus 1.7 times for D/D in *An. gambiae* (*z* = −1.89, *p* = 0.06).

A worrisome observation is that the *Cx pipiens ace-1*^*D1*^ allele totally replaced the local *ace-1*^*R*^ in Martinique^56^. Similarly, *ace-1*^*D*^ appears to be spreading in West Africa. Our study reveals that its higher fitness does not result from a higher resistance level but from a decreased cost, or rather from a new equilibrium between resistance and cost, providing *An. gambiae* mosquitoes with a new evolutionary path.

### *ace-1* ^*D*^ is bad news for malaria vector control in areas with high PYR resistance

According to the results of our study, it is obvious that the selective pressure intensity, in this case the quantity of insecticide used, will be crucial to determine which of *ace-1*^*R*^ or *ace-1*^*D*^ will prevail in treated areas: in highly treated areas, *ace-1*^*R*^ should be favored due to its higher resistance level, while *ace-1*^*D*^ will be fitter in less treated areas, thanks to its lower cost.

However, the heterogeneity of insecticide usage practices could actually be determinant on a larger geographic scale and explain the selection of the duplicated allele in natural populations. Due to the mosaic nature of mosquito control and mosquito’s migration ability, a same individual may experience both treated and untreated areas during its lifespan. This could favor the selection of a more balanced, generalist, phenotypic optimum: a heterozygote individual (R/S) would survive better than R/R individuals in absence of insecticide (lower fitness cost), but also better than S/S individuals in treated areas. Such heterozygote advantage over two contrasted environments is called marginal overdominance^57^. However, heterozygotes cannot become fixed in a population, as the segregation burden leads to the loss of the advantage of having both AChE1S and AChE1R enzymes in half of their progeny. Haldane^58^ proposed that the existence of two functionally divergent alleles leading to overdominance would promote the emergence and selection of a duplication carrying both copies by creating “permanent heterozygotes”. Individuals carrying this duplicated allele would keep their advantage across generations and may invade natural populations. The *ace-1*^*D*^ allele in *An. gambiae* appears as a perfect example of selection for permanent heterozygosity: our study shows that a D/D genotype results in a phenotype similar to a standard heterozygote R/S, but without the segregation burden. Moreover, its distribution in West African natural populations points to the crucial role of the insecticide usage practices (and their heterogeneity) in the selection of this more generalist allele. In Burkina Faso and Ivory Coast, CXs and OPs selective pressures appear moderate but pervasive, as the *ace-1*^*D*^ allele is almost fixed and *ace-1*^*R*^ frequency quite low^23^,^27^,^28^,^53^,^59^. In contrast, both *ace-1*^*R*^ and *ace-1*^*D*^ frequencies are still low in Benin, which certainly reflects a low selective pressure^42^,^60^.

However, while inspiring from a fundamental biology point of view, the spread of *ace-1*^*D*^ in natural populations could represent a serious threat for resistance management strategies. Indeed, such a low-cost resistance allele will be more difficult to root out using classical strategies based on insecticide alternation. Moreover, the currently-deployed American President’s Malaria Initiative in collaboration with the National Malaria Control Program could favor *ace-1* duplication spread if the insecticide pressure is too low or the coverage too heterogeneous. This would select for resistance to the new IRS and ITN, and could be quite disastrous. In high PYR resistance areas, *ace-1*^*D*^ would spread in populations with high frequencies of the *kdr* allele; yet previous studies showed that *kdr* and *ace-1*^*R*^ (*ace-1*^*D*^-kdr interaction has not been investigated so far) act in synergy for both resistance levels and fitness costs^61^,^62^. Furthermore, a recent study showed that *ace-1* duplication associated with enzyme detoxification seems to confer a very high bendiocarb resistance to *An. gambiae* from Ivory Coast^31^. It could thus be reasonable to take beforehand the time to investigate more thoroughly the potential impact of a shift to CXs and OPs for malaria control, particularly in regions where resistance to these insecticides is already present and where *ace-1*^*D*^ is spreading.

## Methods

### Mosquito strains and collection

Mosquito strains: two laboratory strains of *An. gambiae* were used in this study: KisumuP and Acerkis. KisumuP strain was derived from the reference strain Kisumu susceptible to all insecticides^63^. As Kisumu was heterogeneous for two susceptible alleles at the *ace-1* locus, we isolated a new strain, KisumuP, homozygous for a single susceptible allele (*ace-1*^*S*^ allele or S). Acerkis is a strain homozygous for the G119S mutation in *ace-1* gene (*ace-1*^*R*^ allele or R), and resistant to both OPs and CXs insecticides^30^. Both strains mostly share the same Kisumu genetic background.

Mosquito collection: third instar larvae of *An. gambiae* from Baguida (6°09′47″N—1°19′50″E, Togo) were selected with propoxur at 1 mg/L (a concentration killing only S/S individuals) and resistant larvae were reared until adulthood in the laboratory. At the adult stage, we used morphological test and molecular analysis to identify the members of the *An*. *gambiae* complex present^64^,^65^,^66^.

### Acerduplikis strain establishment

#### Fixation protocol

The fixation protocol is illustrated in Supplementary Figure 1. It consists in four successive steps: (A) *Detection of females harboring the ace-1*^*D*^ *allele*: Females emerged from field-collected larvae after propoxur selection were crossed with KisumuP S/S males and then isolated to lay eggs. The offspring of each female was selected with 1 mg/L propoxur. Mothers that displayed offspring with no mortality were phenotyped with the *ace-1* RFLP-PCR test to identify [RS] ones^35^. All these [RS] mothers corresponded either to D/R or D/D genotypes, and thus harbored the duplicated *ace-1*^*D*^ (D) allele. (B) *Elimination of the ace-1*^*R*^ *allele*: Once adult, females were crossed with KisumuP S/S males and were allowed to lay eggs individually. They were then screened with a PCR-RFLP test specific of the D(S) copy. Females identified as D/S genotypes were sequenced for *ace-1* Ex2-7 PCR fragment (see below). As all sequences were found identical, progenies were grouped. (C) *Backcrosses on KisumuP*: These female’s progenies were used for six successive backcrosses with KisumuP males, in order to homogenize the genetic background. (D) *Elimination of the KisumuP ace-1*^*S*^ *allele*: After the last backcross, the strain was crossed on itself and selected with 1 mg/L propoxur for three generations to increase *ace-1*^*D*^ frequency. Progenies were then screened with a PCR test specific of KisumuP. Progenies in which no KisumuP *ace-1*^*S*^ allele were found were then mixed to constitute the Acerduplikis strain, homozygous for the duplicated allele *ace-1*^*D*^, and sharing a genetic background largely similar to KisumuP.

#### Genetic background characterization

After eight backcrosses, most of the Acerduplikis strain was expected to be introgressed by the KisumuP genetic background: at a 5% risk, all the genome except 30 cM around the *ace-1* locus is expected to have recombined^36^. To check this introgression, we developed at least one molecular marker per *An. gambiae* chromosome that was polymorphic between individuals from the KisumuP strain and a mix of ten individuals from the Baguida field population used to establish Acerduplikis (Supp. Table 1). These polymorphic markers were then sequenced on DNA extracted from a mix of about 100 Acerduplikis first-instar larvae.

#### Specific molecular tests

All PCR were performed with 50 ng of genomic DNA in 40 μL final under the following conditions: 94 °C for 30 s, annealing temperature for 30 s, and 72 °C for 1 to 2 min for a total of 33 cycles (primers and annealing temperature are listed in Supp. Table 1).

- *D(S) copy specific PCR-RFLP test*. A PCR using Exon3univdir and AgEx4rev2 primers amplifies a 511 bp fragment from all *An. gambiae ace-1* alleles (Supp. Tab. 1 and Supp. Fig. 2). The restriction enzyme AvaI cuts the *ace-1*^*S*^ and *ace-1*^*R*^ alleles into two fragments (28 bp and 483 bp), and the D(S) copy into three fragments (28 bp, 119 bp and 363 bp). 10 μL of the PCR product were digested with 5 units of enzyme for two hours at 37 °C.

- *KisumuP specific PCR test*. A PCR using Kisumudir2 and Kisumurev1 primers is specific to the KisumuP *ace-1*^*S*^ allele; none of the other *ace-1* alleles present was amplified (Supp. Fig. 2).

#### *ace-1* ^*D*^ sequencing

Genomic DNA from single mosquitoes was amplified using the AgEx2dir1 and AgEx7rev2 primers (2241 bp PCR fragment, from exon 2 to exon 7 (Supp. Fig. 2). PCR products were purified using the QIAquick Gel Extraction Kit (QIAGEN). For the KisumuP and Acerkis strains, the purified PCR product was directly sequenced. For Acerduplikis, the purified PCR product was cloned using the TOPO TA Cloning^®^ kit following to the manufacturer instructions (Invitrogen Life Science Technologies), to separate the different duplicated copies, D(S) or D(R). The clones were screened for the presence of the G119S substitution, and at least six clones were sequenced for each copy. Sequencing was conducted on an ABI Prism 310 sequencer (BigDye Terminator Kit, Applied Biosystems, Foster City, CA). Each clone was sequenced using the primers AgEx2dir1 and AgEx7rev2, plus an internal primer due to the fragment length, AgIntdir1 (Supp. Fig. 2). Exon 2 to exon 7 sequences of the susceptible single-copy allele from KisumuP (Ag-*ace-1*^*S*^), of the resistant single-copy allele from AcerKis (Ag-*ace-1*^*R*^) and of the susceptible (Ag-*ace-1*^*D*^-S) and resistant (Ag-*ace-1*^*D*^-R) copies of the duplicated allele were deposited in GenBank (accession numbers KM875634, KM875637, KM875635 and KM875636, respectively).

### *ace-1* duplication mapping

Only the strains KisumuP and Acerduplikis were used at this stage.

- *Chromosomes preparation*. Ovaries were pulled out from ~4 days-old half-gravid females, 25 hours post blood-feeding, at Christopher’s Stage III of development^67^, and preserved in fresh Carnoy’s fixative solution (3 volume ethanol: 1 volume glacial acetic acid). Ovaries were fixed for 24 h at room temperature and stored at −20 °C. Polytene chromosome slide preparation was performed as described by Sharakhova *et al.*^68^.

- *Probes preparation*. Probe1 was specific to the *ace-1* gene and probe2 was specific of the AGAP001373 gene, located about 500 kb from *ace-1* on chromosome 2R in *An. gambiae* genome (https://www. vectorbase.org/Anopheles gambiae). Using KisumuP DNA, the probe1 2241 bp fragment was amplified with AgEx2dir1 and AgEx7rev2 primers and the probe2 1861 bp fragment was amplified with Ag0.5MBdir2 and Ag0.5MBrev2 primers (Supp. Table 1). These fragments were cloned with TOPO TA Cloning^®^ kit following the manufacturer instructions (Invitrogen Life Science Technologies). DNA probes were labelled separately with DIG-Nick Translation Mix (Digoxigenin-11-dUTP) according to the manufacturer Protocol (Roche Diagnostics). Hybridization and detection followed a previously described procedure^69^. Fluorescent signals were recorded using a Zeiss Axiophot microscope equipped with phase-contrast and fluorescence image analyzer (Cytovision 3.93.2). Three polytene chromosome slides were hybridized for each strain and each probe then co-hybridized for Acerduplikis strain with probe1 and probe2.

### Acerduplikis *ace-1* copy number quantification

The number of *ace-1* gene copies was estimated relatively to a reference gene AGAP010592 = AgRps7 (found in a single copy in the Pest strain genome, Vector Base https://www.vectorbase.org/) by Real-time quantitative PCR performed with a LC480 Light Cycler (Roche). Two PCRs were performed on each DNA, one specific of *ace-1* locus (Agace1qtidir2 and Agace1qtirev2 primers) and the other specific of the reference gene (AgS7Ex5qtidir and AgS7Ex5qtirev primers) (Supp. Table 1). 1 ng of each genomic DNA (normalised with the Qubit 2.0 Fluorometer-Invitrogen) was mixed with 0.6 μM or 0.8 μM of *ace-1* or *Rps7* specific primers respectively and 3 μL of mastermix (LightCycler 480 SYBR Green, Roche). PCR was performed with a 95 °C activation step for 8 min followed by 45 cycles of 95 °C for 4 s, 67 °C for 13 s, and 72 °C for 19 s. Each DNA template was analyzed in four replicates for both genes. The ratio between *ace-1* and *Rps7* arbitrary concentrations was determined with the Advanced Relative Quantification method of the LightCycler 480 software 1.5.0.

### Bioassays with Carbamate and Organophosphate insecticides

Resistance data for the three strains (KisumuP, Acerkis and Acerduplikis) and their F1 offspring (*ace-1* genotypes R/S (Acerkis x KisumuP), D/S (Acerduplikis x KisumuP) and D/R (Acerduplikis x Acerkis)) were compared. Five insecticides of technical grade quality were used, one CX: bendiocarb (99.5% pure), three OPs: chlorpyrifos methyl (99.9% pure), fenitrothion (95.2% pure) and dichlorvos (98.9% pure), and one PYR: permethrin (98.3% pure). Insecticide solutions were prepared in 70% ethanol and stored at 4 °C in a dark room to avoid photolysis. A set of 25 late third- and early fourth-instar larvae was incubated in 99 ml of distilled water in plastic cups, to which 1 ml of insecticide solution at the required concentration was added. Four replicates were performed for each concentration. Six to twelve insecticide concentrations providing a range of mortality from 0 to 100% were used for each insecticide tested. Larval mortality was recorded after a 24 hours exposure. Control bioassays were performed by adding 1 ml of ethanol to 99 ml of distilled water. Temperature was maintained at 27 °C ± 2 °C during bioassays (temperature measured using Waranet technology, Waranet Solutions SAS, Auch, France).

The analyses of dose-mortality responses in bioassays were performed using the R software (v.3.0.0). The R script BioRssay (v.6.1;^70^) was used; it is freely available on the website of the Institut des Sciences de l’Evolution de Montpellier. This script computes the doses of insecticide killing 50% and 95% of the tested population or strain (Lethal Concentration 50 and 95, or LC_50_ and LC_95_) and the associated confidence intervals, and tests for the linearity of the dose-mortality response (χ^2^ test). Finally, it allows the comparison of two or more strains or populations and calculates the resistance ratios, i.e. RR_50_ or RR_95_ (=LC_50_ or LC_95_ of tested population/LC_50_ or LC_95_ of the reference strain, resp.) and their 95% confidence intervals.

### Fitness cost parameters

#### Larval mortality and development time

To assess the development time and pre-imaginal mortality associated with different *ace-1* alleles, assays were performed as described by Agnew *et al.*^71^. Females’ oviposition was synchronized for the three strains. At egg hatching, 96 first-instar larvae from each strain were individually transferred to *Drosophila* tubes for rearing in 1ml of mineral water at 2 g/L concentration of TetraMin® powdered fish food (Tetramin BabyMin, Tetra Gmbh, Melle, Germany). Food was provided once, the first day of experiment. Tubes were arranged on racks and maintained in insectary conditions (27 ± 2 °C, 80 ± 2 humidity, 12 h: 12 h light:dark). The racks were randomly moved every day to reduce positional effects. Dead larvae or pupae were counted every day to assess the mortality rate at each development stage. Timing of adult emergence was also recorded.

#### Mating competition

Virgin adults (two-day old) reared under laboratory standard conditions were crossed in cages (30 cm × 30 cm × 30 cm). Trials were performed between two males of each competing genotype (S/S *vs* R/R, S/S *vs* D/D or D/D *vs* R/R) placed in the presence of either ten S/S or ten D/D females. Each competition cage was replicated ten times. Mosquitoes had access *ad libitum* to a honey solution. After three days, females were blood-fed on rabbit and allowed to lay eggs individually. After hatching, each female progeny was selected with an insecticide dose that allows paternity assignation. When females were S/S, paternity in the S/S *vs* R/R and S/S *vs* D/D trials was assigned with propoxur at 1 mg/L (which kills only S/S progeny); in the D/D *vs* R/R trial, paternity was assigned with bendiocarb at 1 mg/L (which kills D/S but not R/S progeny). When females were D/D, paternity in the S/S *vs* R/R and S/S *vs* D/D trials was assigned with bendiocarb at 1 mg/L, while paternity in the DD *vs* RR trial was assigned with bendiocarb at 5 mg/L (which kills D/D but not D/R progeny). The paternity success of a given genotype was defined in each replicate of trial as the percentage of egg-rafts it had sired.

#### Female fecundity and fertility

All strains were reared under the same soft environmental conditions and crosses were performed between 200 males and 200 females. After at least three days, females were blood-fed and 40 gravid females from each strain were allowed to oviposit individually in plastic cups containing 70 mL dechlorinated water. Three days after blood feeding, the number of egg-laying females and the amount of eggs per female were recorded. Two days after, the number of hatching larvae per female was counted.

### Statistical analyses

*ace-1* gene copy number variation among S/S and D/D genotypes was analyzed using linear models. Normality of the model residuals and homoscedasticity were checked using Shapiro-Wilk and Breusch-Pagan tests, respectively^70^.

Larval mortality and development time were analyzed using Cox proportional hazards regression model^70^.

Differences in paternity success between trails were tested using generalized linear models (GLM), with a binomial error distribution. Departure from the expected proportion of 0.5 within each trial (*i.e.* if the two male genotypes display the same ability to fecund females) was then tested using exact binomial tests^70^.

Differences among genotypes in the rate of females laying eggs and in the hatching rate were tested using GLM with binomial error distributions. Differences among genotypes in egg numbers and larvae numbers per female were tested using GLM with Gaussian error distributions^70^.

All computations were performed using the R free software (v.3.1.1, http://www.r-project.org). Cox’s models and GLM were simplified as follow: significance of the different terms was tested starting from the higher-order terms using likelihood ratio test (LRT). Non-significant terms (*p* > 0.05) were removed^72^. Factor levels of qualitative variables that were not significantly different were grouped (LRT^72^).

## Additional Information

**How to cite this article**: Assogba, B. S. *et al.* An *ace-1* gene duplication resorbs the fitness cost associated with resistance in *Anopheles gambiae*, the main malaria mosquito. *Sci. Rep.* **5**, 14529; doi: 10.1038/srep14529 (2015).

## Supplementary Material

Supplementary Information

## Figures and Tables

**Table 1:** Dose-mortality responses to different insecticides observed in reference strains of *Anopheles gambiae* s. s.

*ace-1* genotypes	Insecticides														
	Bendiocarb			Chlorpyrifos-methyl			Fenitrothion			Dichlorvos			Permethrin		
	aLC_50_(mg/L)	bRR_50_	cChi(*p*)	aLC_50_(mg/L)	bRR_50_	cChi(*p*)	aLC_50_(mg/L)	bRR_50_	cChi(*p*)	aLC_50_(mg/L)	bRR_50_	cChi(*p*)	aLC_50_(mg/L)	bRR_50_	cChi(*p*)
S/S (Kisumu)	0.22	NA	0.99	0.004	NA	0.99	0.003	NA	0.99	0.008	NA	1	0.006	NA	0.99
R/R (Acerkis)	50.1	229.3	0.99	0.036	9.04	1	0.061	23.74	0.99	0.096	12.61	0.99	0.006	1	0.99
R/S	27.04	123.9	0.99	0.007	1.72	0.99	0.021	8.39	0.91	0.05	6.4	0.99	NA	NA	NA
D/S	0.28	1.29	1	0.006	1.56	0.99	0.016	6.28	0.99	0.04	5.4	0.99	NA	NA	NA
D/R	26.96	123.5	0.99	0.013	3.21	0.99	0.042	16.57	0.99	0.06	7.62	0.99	NA	NA	NA
D/D (Acerduplikis)	0.68	3.14	1	0.007	1.91	0.99	0.022	8.78	0.99	0.05	6.56	1	0.006	1	0.99

^a^LC_50_: lethal concentration in milligrams per liter inducing a mortality of 50%.

^b^RR_50_: resistance ratio at LC_50_ = LC_50_(resistant strain)/LC_50_(Kisumu).

^c^Chi(*p*): the *p*-value of chi-square test for linearity of the dose response; *p*-values > 0.05 indicate acceptable fits (i.e. linearity is not rejected).
