# Supplementary material for: An ace-1 gene duplication resorbs the fitness cost associated with resistance in Anopheles gambiae, the main malaria mosquito
Source: Sci Rep. 2015 Oct 5;5:14529. doi: 10.1038/srep14529 (PMC4592963; doi:10.1038/srep14529)
Supplement: Supplementary Information [file srep14529-s1.pdf]

# *An ace-1 gene duplication resorbs the fitness cost associated with resistance in Anopheles gambiae, the main malaria mosquito*

Benoît S. Assogba, Luc S. Djogbénou, Pascal Milesi, Arnaud Berthomieu, Julie Perez, Diego Ayala, Fabrice Chandre, Michel Makoutodé, Pierrick Labbé, Mylène Weill.

## Supplementary information

**Supplementary Figure 1: Acerduplikis strain fixation protocol.** See Material and Methods text for details.

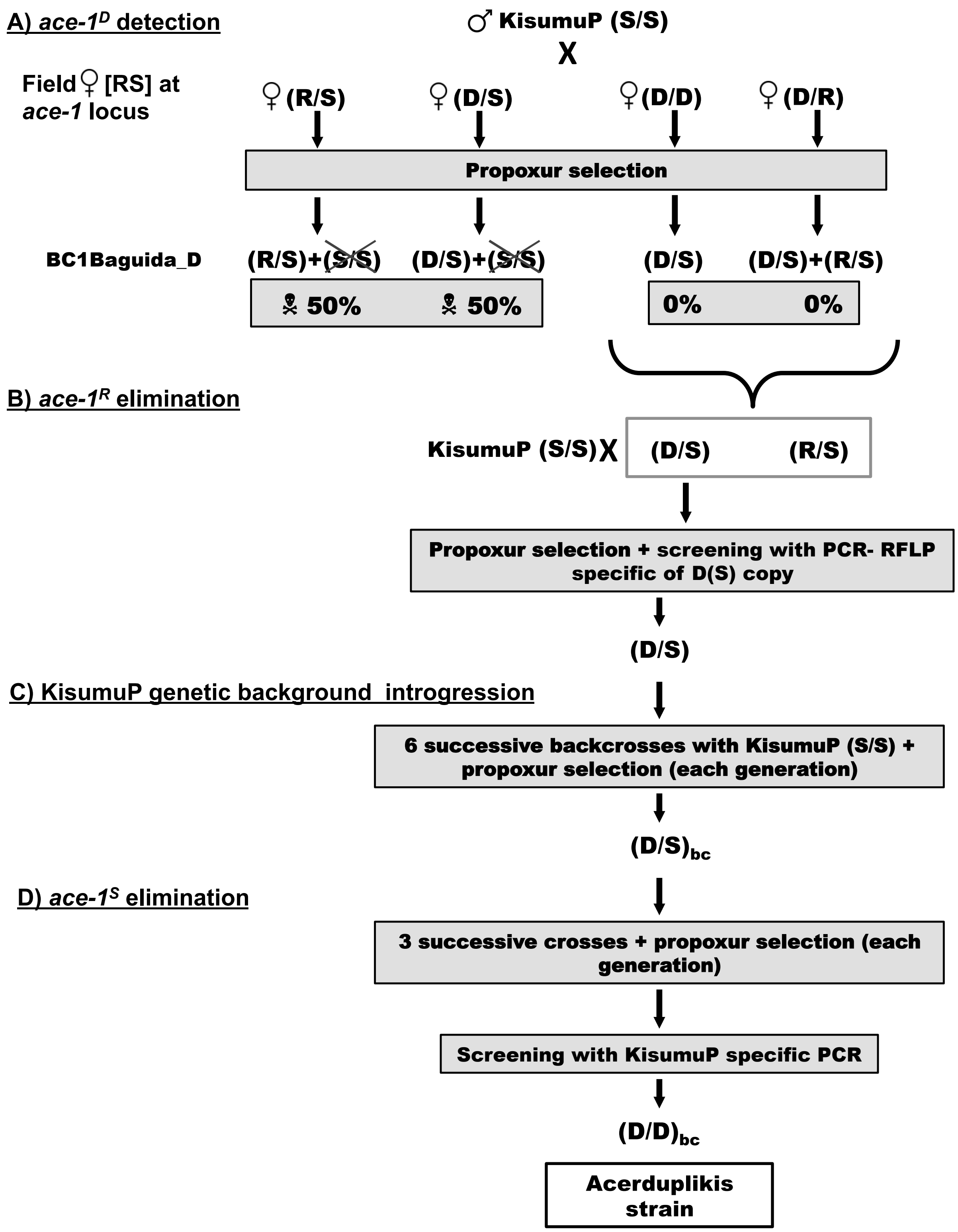

**Supplementary Figure 2: Sequences alignment from exon 2 to exon 7 of *ace-1*$^S$ allele from Kisumu, D(S) and D(R) copies of *ace-1*$^D$ allele from Acerduplikis, and *ace-1*$^R$ resistant allele from Acerkis.** The G119S mutation in box. The primers used for this study are highlighted in grey. AvaI restriction enzyme sites are highlighted in black. Only polymorphic sites with the top sequence are indicated and dash indicates deletions.

```
               AgEx2dir1                                                                            100
ace-1S    AGGTCACGGTGAGTCCGTACGAATTATAGATGCCGAGTTGGGCACGCTCGAGCATGTCCACAGTGGAGCAACGCCGCGGCGACGCGGTCTGACGAGGCGC
D(S)      .............................................A.........................................C............
ace-1R    ....................................................................................................
D(R)      ....................................................................................................
                                                                                                        200
ace-1S    GAGTCCAACTCGGGTAAGTACGCGATTGGAAGTGGGGGGACGTTTACCCTGCCGTGTACTACAATGCACTTTACCCCCACGCACACGCACCGGCAGACGC
D(S)      .....A............................................A.............C...................................
ace-1R    .....A............................................A.............C...................................
D(R)      .....A............................................A.............C...................................
                                                                                                        300
ace-1S    GAACGACAACGATCCGCTGGTGGTCAACACGGATAAGGGGCGCATCCGCGGCATTACGGTCGATGCGCCCAGCGGCAAGAAGGTGGACGTGTGGCTCGGC
D(S)      ................................................................................................T...
ace-1R    ....................................................................................................
D(R)      ....................................................................................................
                    Kisumudir2                                                                          400
ace-1S    ATTCCCTACGCCCAGCCGCCGGTCGGCCGTTACGGTTCCGTCATCCGCGGCCGGCCGAAAAGTGGACCGGCGTGCTGAACACGACCACACCGCCCAACA
D(S)      ..........................C...C.....................................................................
ace-1R    ..............................C.....................................................................
D(R)      ..............................C.....................................................................
                    Exon3univdir          AvaI            AgAce1qtidir2                                 500
ace-1S    GCTGCGTGCAGATCGTGGACACCGTGTTCGGCGACTTCCCGGGCGCGACCATGTGGAACCCGAACACGCCCCTGTCCGAGGACTGTCTGTACATTAACGT
D(S)      ....................................................................................................
ace-1R    ....................................................................................................
D(R)      ....................................................................................................
                                                                                                        600
ace-1S    GGTGGCACCGCGACCCCGGCCCAAGAATGCGGCCGTCATGCTGTGGATCTTCGGCGGC[G]GCTTCTACTCCGGCACCGCCACCCTGGACGTGTACGACCAC
D(S)      ..........................................................[.].........................................
ace-1R    ............G.............................................[A].........................................
D(R)      ............G.............................................[A].........................................
                         AgAce1qtirev2                                                                  700
ace-1S    CGGGCGCTTGCGTCGGAGGAGAACGTGATCGTGGTGTCGCTGCAGTACCGCGTGGCCAGTCTGGGCTTCCTGTTTCTCGGCACCCCGGAAGCGCCGGGCA
D(S)      ....................................................................................................
ace-1R    ......................................................................................C.............
D(R)      ......................................................................................C.............
                                                        Kisumurev1                  AgIntdir1           800
ace-1S    ATGCGGGACTGTTCGATCAGAACCTTGCGCTACGGTAGGTGTCTTTGCATGGGTGAATGAGGGTATAGTATTCTAACGAGGTGCTCTTCTTCCCATCACT
D(S)      ...................................................T....G...CCC.T...................................
ace-1R    ................................................G..T...TC..TA.T.....................................
D(R)      ................................................G..T...TC..TA.T.....................................
            AvaI                                                                                        900
ace-1S    TCTTGGGAGTCAGCTGGGTGCGGGACAACATTCACCGGTTCGGTGGTGATCCGTCGCGTGTGACACTGTTCGGCGAGAGTGCCGGTGCCGTCTCGGTGTC
D(S)      ...C.................................................................................C..............
ace-1R    ..........................................................C.........................................
D(R)      ..........................................................C.........................................
              AgEx4rev2                                                                                 1000
ace-1S    GCTGCATCTGCTGTCCGCCCTGTCCCGCGATCTGTTCCAGCGGGCCATCCTGCAGAGCGGCTCGCCGACGGCACCGTGGGCATTGGTATCGCGCGAGGAA
D(S)      .....................T..............................................................................
ace-1R    .....................T..............................................................................
D(R)      .....................T..............................................................................
                                                                                                        1100
ace-1S    GCCACGCTAAGGTACGTGCCAGCTGCTGCTTTCCCCAAACCACCAACCCGCGACAGCTCACACAACCCTCTTTTCCTTCGCTCTTTTCTCGCTCCAGAGC
D(S)      ..................T..........G...........G..........................A...............................
ace-1R    ...................................................A........................G.......................
D(R)      ...................................................A........................G.......................
                                                                                                        1200
ace-1S    ACTGCGGTTGGCCGAGGCGGTCGGCTGCCCGCACGAACCGAGCAAGCTGAGCGATGCGGTCGAGTGTCTGCGCGGCAAGGATCCGCACGTGCTGGTCAAC
D(S)      .................................................................................C..................
ace-1R    ....................................................................................................
D(R)      ....................................................................................................
                                                                                                        1300
ace-1S    AACGAGTGGGGCACGCTCGGCATTTGCGAGTTCCCGTTCGTGCCGGTGGTCGACGGTGCGTTCCTGGACGAGACGCCGCAGCGTTCGCTCGCCAGCGGGC
D(S)      ....................................................................................................
ace-1R    ....................................................................................................
D(R)      ....................................................................................................
```

```
                                                                                                              1400
ace-1^S   GCTTCAAGAAGACGGAGATCCTCACCGGCAGCAACACGGAGGAGGGCTACTACTTCATCATCTACTACCTGACCGAGCTGCTGCGCAAGGAGGAGGGCGT
D(S)      ....................................................................................................
ace-1^R   ....................................................................................................
D(R)      ....................................................................................................
                                                                                                              1500
ace-1^S   GACCGTGACGCGCGAGGAGTTCCTGCAGGCGGTGCGCGAGCTCAACCCGTACGTGAACGGGGCGGCCCGGCAGGCGATCGTGTTCGAGTACACCGACTGG
D(S)      ....................................................................................................
ace-1^R   ....................................................................................................
D(R)      ....................................................................................................
                                                                                                              1600
ace-1^S   ACCGAGCCGGACAACCCGAACAGCAACCGGGACGCGCTGGACAAGATGGTGGGCGACTATCACTTCACCTGCAACGTGAACGAGTTCGCGCAGCGGTACG
D(S)      ....................................................................................................
ace-1^R   ....................................................................................................
D(R)      ....................................................................................................
                                                                                                              1700
ace-1^S   CCGAGGAGGGCAACAACGTCTACATGTATCTGTACACGCACCGCAGCAAAGGCAACCCGTGGCCGCGCTGGACGGGCGTGATGCACGGCGACGAGATCAA
D(S)      ....................................................................................................
ace-1^R   ....................................................................................................
D(R)      ....................................................................................................
                                                                                                              1800
ace-1^S   CTACGTGTTCGGCGAACCGCTCAACCCCACCCTCGGCTACACCGAGGACGAGAAAGACTTTAGCCGGAAGATCATGCGATACTGGTCTAACTTTGCCAAA
D(S)      ....................................................................................................
ace-1^R   ....................................................................................................
D(R)      ....................................................................................................
                                                                                                              1900
ace-1^S   ACCGGGTAAGTGTGTGTGTGTGTGTGTGTCAAACAGCAGAGTGTCGATCGCTCTAACGCC------TTCTCTCTTCAACAGCAATCCAAATCCCAACACG
D(S)      ...................----------.........A....C.A..A........A..AGCGTC..........T......................A
ace-1^R   ...................----------.........A....C.A..A........A..AGCGTC..........T......................A
D(R)      ...................----------.........A....C.A..A........A..AGCGTC..........T......................A
                                                                                                              2000
ace-1^S   GCCAGCAGCGAATTCCCCGAGTGGCCCAAGCACACCGCCCACGGACGGCACTATCTGGAGCTGGGCCTCAACACGTCCTTCGTCGGTCGGGGCCCACGGT
D(S)      ....................................................................................................
ace-1^R   ....................................................................................................
D(R)      ....................................................................................................
                                                                                                              2100
ace-1^S   TGAGGCAGTGTGCCTTCTGGAAGAAGTACCTTCCCCAGCTAGTTGCAGCTACCTGTAAGTCTAGTTGCTGCGCGAGAAACCCCCTCCTCTCGCGTCCCCA
D(S)      .......................................................................A..............---...........
ace-1^R   .......................................................................A..............---...........
D(R)      .......................................................................A..............---...........
                                                                                                              2200
ace-1^S   TCAGGGTCCAGGTTGCAATAACAAATGTATCTCTCTCTCACGTCTCCTTTTTCTCCAAAACAGCGAACCTACCAGGGCCAGCACCGCCCAGTGAACCGTG
D(S)      ...........A..A............................A..--.....C..............................................
ace-1^R   ...........A..A............................A..--.....C..............................................
D(R)      ...........A..A............................A..--.....C..............................................
                                      AgEx7rev2   2241
ace-1^S   CGAAAGCAGCGCATTTTTTTACCGACCTGATCTGATCGTGC
D(S)      .........................................
ace-1^R   .........................................
D(R)      .........................................
```

**Supplementary Figure 3**: ***ace-1* copy number quantification in Acerduplikis (DD) and KisumuP (SS) strains.** Dots indicate the ratios observed in each trial between *ace-1* and *Rps7* genes concentrations determined with Advanced Relative Quantification method of the LightCycler 480 software 1.5.0.

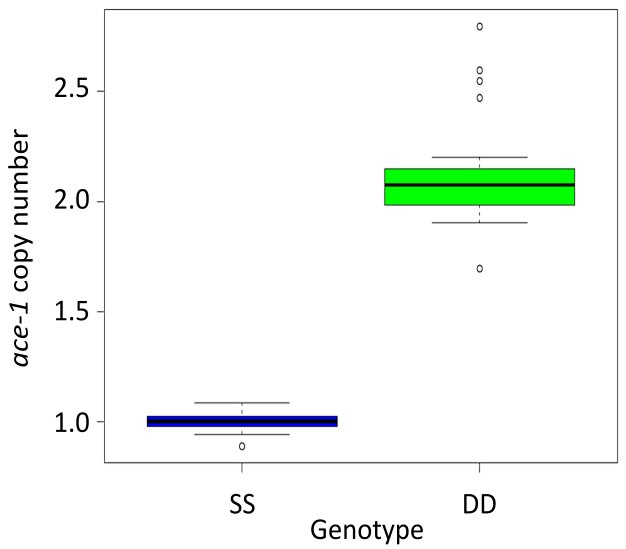

**Supplementary Figure 4: OP insecticide bioassays**. The mortality in relation to the insecticide dose is presented for six genotypes (see legend below). A, B, C and D panels correspond respectively to bendiocarb, chlorpyrifos-methyl, fenitrothion and dichlorvos insecticide. Graphs were drawn using the BioRssay script (Milesi et al. 2013).

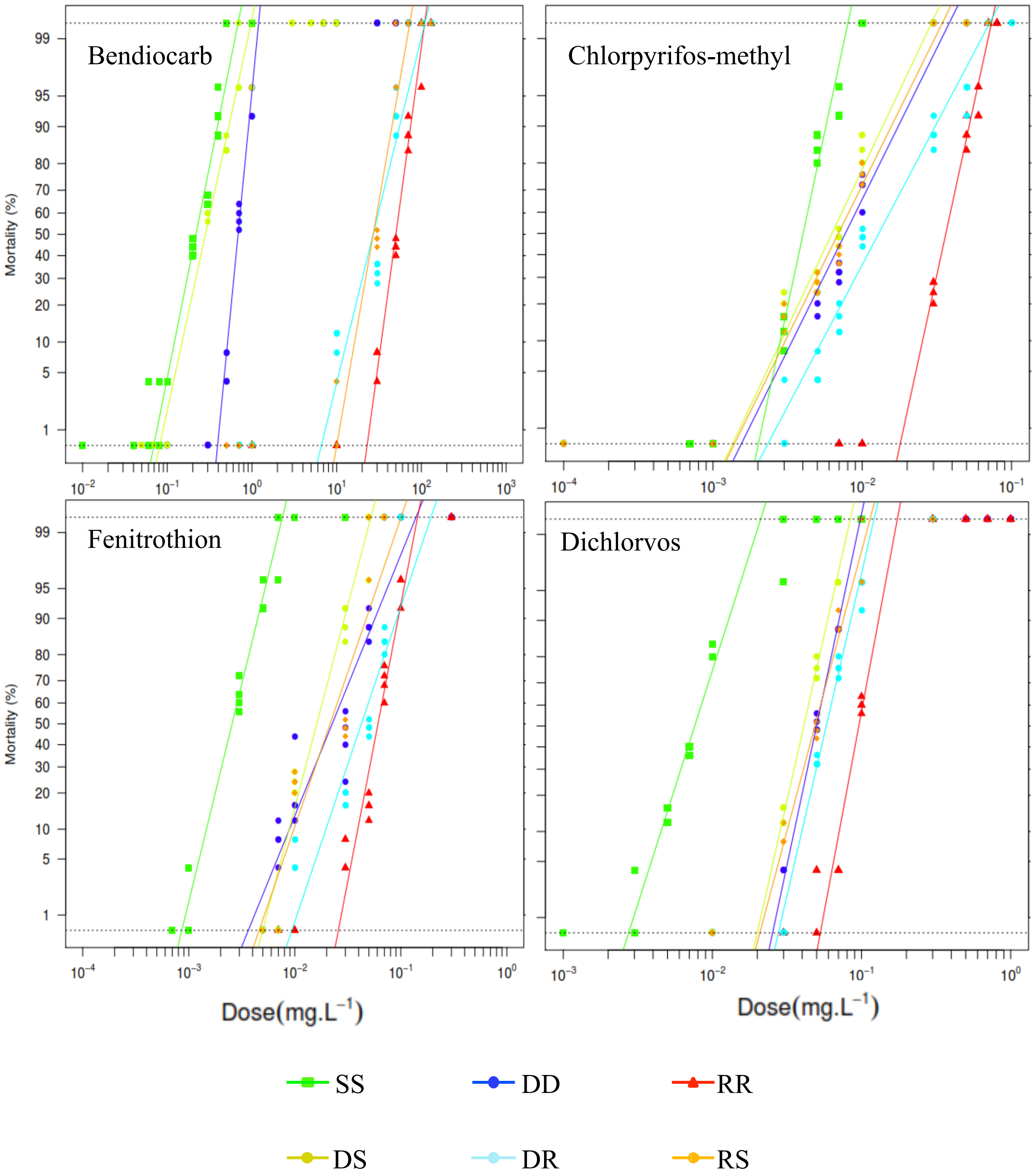

**Supplementary Figure 5: Female fertility and fecundity for the susceptible (SS), resistant (RR) and duplicated (DD) homozygotes.** (A) Average egg-laying rates (i.e. the number of females that laid eggs over the total number of females assayed) with their 95% confidence intervals (CI), (B) the average eggs number per female with the associated standard deviations (SD). (C) the average hatching rates (i.e. the number of larvae produced over the number of eggs in the raft) with the associated SD and (D) the average larvae number by egg-laying females, with the associated SD, are presented for SS (green), DD (blue) and RR (red). Significance of the differences among the various genotypes is indicated (n.s., $p > 0.05$; *, $p < 0.05$; **, $p < 0.01$; ***, $p < 0.001$).

*Analysis*: The rate of egg-laying females (ELF) among forty (A) was not impacted by their genotype (GLM ELF = GENO + $\varepsilon$ (binomial distribution), $\chi^2 = 0.46$, $\Delta df = 2$, $p = 0.79$). However, R/R females laid each significantly less eggs (54.3±24) than D/D or S/S females (respectively, 75.8±34 and 86.1±37; B) (GLM NEGGS = GENO + $\varepsilon$ (gaussian distribution), $F = 7.63$, $\Delta df = 2$, $p < 0.001$); the difference between D/D and S/S was not significant ($F = 2.86$, $\Delta df = 1$, $p = 0.09$). As the hatching rate (HR, C) was not significantly different between genotypes (GLM HR = GENO + $\varepsilon$ (binomial distribution), $\chi^2 = 0.01$, $\Delta df = 2$, $p = 0.87$), this resulted in significantly reduced numbers of larvae hatching (NLH) in the progenies of females R/R (24.6±22) as compared to the progenies of females D/D and S/S (36.8±21.9 and 44.3±36; D) (GLM NHL = GENO + $\varepsilon$ (gaussian distribution), $F = 4.63$, $\Delta df = 2$, $p < 0.05$); the difference between D/D and S/S was again not significant ($F = 2.83$, $\Delta df = 1$, $p = 0.1$).

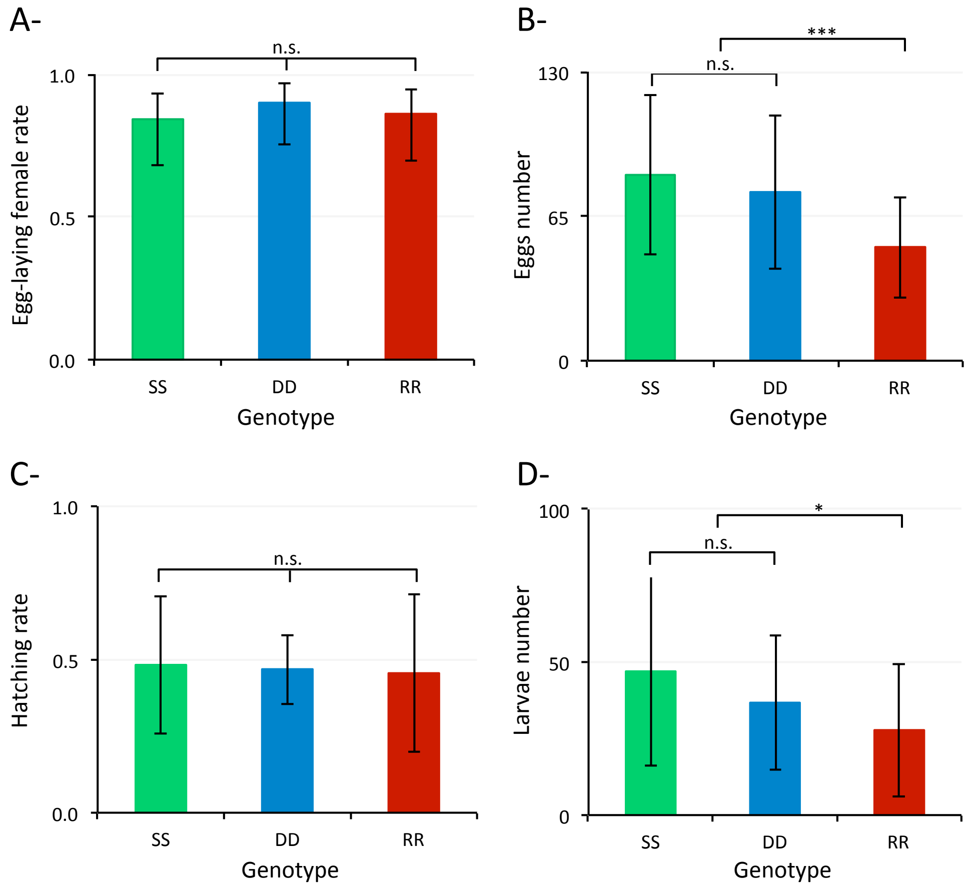

**Supplementary Table 1: List of the primers used in this study and amplicon sizes.**

| Marker name | Locus | Chromosome location | Primer (5'-3') | Size (bp) | Tm (°C) |
|---|---|---|---|---|---|
| *Chromosome markers* | | | | | |
| X2Ag | AGAP000151 | X | X2Ag_Dir: TTGAGAGCGAAAGTTCTGAGC<br>X2Ag_Rev: AACCCCTCCTTTTAAACTTCC | 300 | 52 |
| 2R14Ag | AGAP002049 | 2R | 2R14Ag_Dir: GTTTGGGCGCAACGAAA<br>2R14Ag_Rev: CACCAGCCAGTGTTTCCA | 247 | 52 |
| 2R55Ag | AGAP004383 | 2R | 2R55Ag_Dir: AAAAGCTGGGCATCACGTT<br>2R55Ag_Rev: AAGCTCACCGGTAACACGAG | 562 | 52 |
| 3R28Ag | AY063776.1 | 3R | 3R28Ag_Dir: TGCCGAAACCTGTGCTATAC<br>3R28Ag_Dir: GCACCGTTGATCTCCTCATAG | 754 | 52 |
| *Specific molecular tests* | | | | | |
| D(S) copy | *ace-1* | 2R | Exon3univdir: GATCGTGGACACCGTGTTCG<br>AgEx4rev2: GGGCGGACAGCAGATGCAGCGA | 511 | 58 |
| KisumuP | *ace-1* | 2R | Kisumudir2: AGCCGCCGGTCGGGCCGT<br>Kisumurev1: CTATACCCTCATTCACCCAT | 464 | 61 |
| *Sequencing* | | | | | |
| | *ace-1* | 2R | AgEx2dir1: AGGTCACGGTGAGTCCGTACGA<br>AgEx7rev2: GCACGATCAGATCAGGTCG<br>+ AgIntdir1 (intermediate primer for sequencing): AGGTGCTCTTCTTCCCATC | 2241 | 58 |
| *Fish Probe* | | | | | |
| | AGAP001373 | 2R | Ag0.5MBdir2: GATTCGGCTGGTCGTAACTA<br>Ag0.5MBrev2: ATTTCGCCGAATGAAATCTG | 1861 | 58 |
| *qPCR* | | | | | |
| | *ace-1* | 2R | Agace1qtidir2 : ATGTGGAACCCGAACACG<br>Agace1qtirev2 : ACCACGATCACGTTCTCCTC | 185 | 67 |
| | AGAP010592 | 3L | AgS7Ex5qtidir: GTGTACAAGAAGCTGACTGGC<br>AgS7Ex5qtirev : TAGCTGCTGCAAACTTCGG | 107 | 67 |
